# Infectious Spondylitis Caused by Streptococcus gordonii

**DOI:** 10.7759/cureus.36657

**Published:** 2023-03-24

**Authors:** Weon-min Cho, Ju-sung Lee, Seung-Won Chung, Tae-Keun Ahn

**Affiliations:** 1 Department of Orthopedic Surgery, CHA Bundang Medical Center, CHA University School of Medicine, Sungnam, KOR; 2 Department of Dentistry, CHA Bundang Medical Center, Sungnam, KOR; 3 Department of Orthopedic Surgery, CHA University, CHA Bundang Medical Center, Sungnam, KOR

**Keywords:** spine surgury, lumbar-fusion, lumbar spinal drainage, oral infection, spine injury

## Abstract

Infectious spondylitis is a rare but severe disease of the spine caused by bacteria or other pathogens. Particularly in immunocompromised patients, a definitive source of infection often remains uncertain. Among many pathogens,* Streptococcus gordonii, *a normal oral flora, is a very rare pathogen in infectious spondylitis. Only a few articles have reported infectious spondylitis caused by* Streptococcus gordonii. *To the best of our knowledge, there have been no reports of surgically treated infectious spondylitis caused by *Streptococcus gordonii*. Hence, in the current report, we present the case of a 76-year-old woman with known type 2 diabetes who was transferred to our medical center due to infectious spondylitis caused by *Streptococcus gordonii* following an L1 compression fracture and underwent an operation for treatment.

## Introduction

Infectious spondylitis accounts for 5% of all skeletal infections. It is a serious disease with a mortality rate of 1-20%, depending on patients’ general conditions and causative organisms [1,2]. Infectious spondylitis is grossly classified as a pyogenic infection caused by bacteria or a granulomatous infection caused by mycobacterium or fungus. The incidence of infectious spondylitis has increased by 150% since the 1990s [3], likely due to increases in spine surgeries and the number of patients with medical risk factors such as diabetes, obesity, immunosuppression, and old age. Among the causative bacteria of pyogenic spondylitis, Staphylococcus aureus is the most common, followed by Escherichia coli. However, a definitive source of infection remains uncertain in up to 52% of patients with pyogenic spondylitis [4].

According to the literature on pyogenic spondylitis, only 2% of infections originate from the oral cavity [4]. However, the role of the oral environment in this disease may be underestimated. To date, only a few oral flora microorganisms, such as Aggregatibacter actinomycetemcomitans and Porphyromonas gingivalis, have been shown to be associated with both periodontitis and systemic infection [5,6]. In this paper, the authors report a rare case of infectious spondylitis caused by Streptococcus gordonii, a normal oral flora.

## Case presentation

A 76-year-old woman with known hypertension, type 2 diabetes, and hypothyroidism was transferred to our medical center. She had been diagnosed with an L1 compression fracture two weeks ago and treated conservatively. Despite three fluoroscopy-guided injections at a local clinic for pain associated with a previous L1 compression, the back pain did not improve. Upon arrival at our medical center, she exhibited general weakness in the absence of fever and increased inflammatory markers, including C-reactive protein, erythrocyte sedimentation rate, and a high leukocyte count. The patient showed no signs of an upper respiratory infection, urinary tract infection, or other infections, though she complained of intermittent bleeding from her teeth. The patient had undergone dental prosthetic treatment six months before the lumbar fracture.

Her neurological examinations were normal. Perianal sensation and anal tone were intact. There was tenderness on palpation of her lumbar spine at the fracture site but no evidence of skin or soft tissue infection. Her laboratory findings were as follows: C-reactive protein, 20.84 mg/dL; white blood cell count, 10.48 × 10^3^/mL; absolute neutrophil count (ANC), 84.9%; erythrocyte sediment rate, 74 mm/hour; procalcitonin, 0.271; HbA1c level, 9.3; creatinine, 0.60 mg/dL; and serum glucose, 325 mg/dL. Culture results from blood, sputum, and urine showed no bacterial growth (Table 1).

**Table 1:** Initial lab results

Serum CRP	20.84 mg/dL	(0−0.3)	Urine glucose	3+	
Serum WBC	10.48 × 10^3^/µL	(3.7−9.8)	Urine protein	+-	
Seg	84.9%	(30−75)	Urine leukocyte	-	
Serum Hgb	10.4 g/dL	(11−16)	Urine WBC	<1/HPF	(0−1)
Serum Na	127 mEq/L	(135−145)	Urine bacteria	None/HPF	
Serum creatinine	0.60 mg/dL	(0.5−0.9)	HbA1c	9.3%/T Hb	(4.0−6.0)
Serum glucose	325 mg/dL	(74−109)			

**Figure 1:**
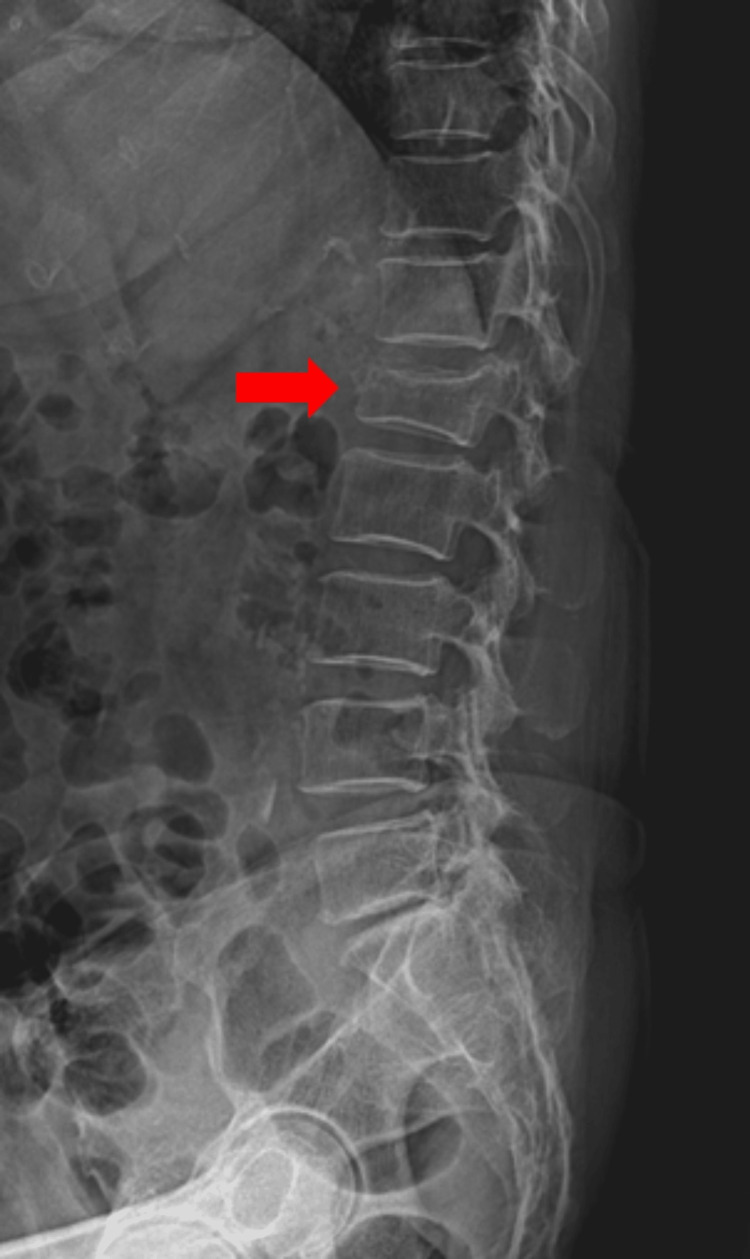
Sagittal plain radiograph of lumbar spine: compression fracture of L1 vertebral body (red arrow).

**Figure 2:**
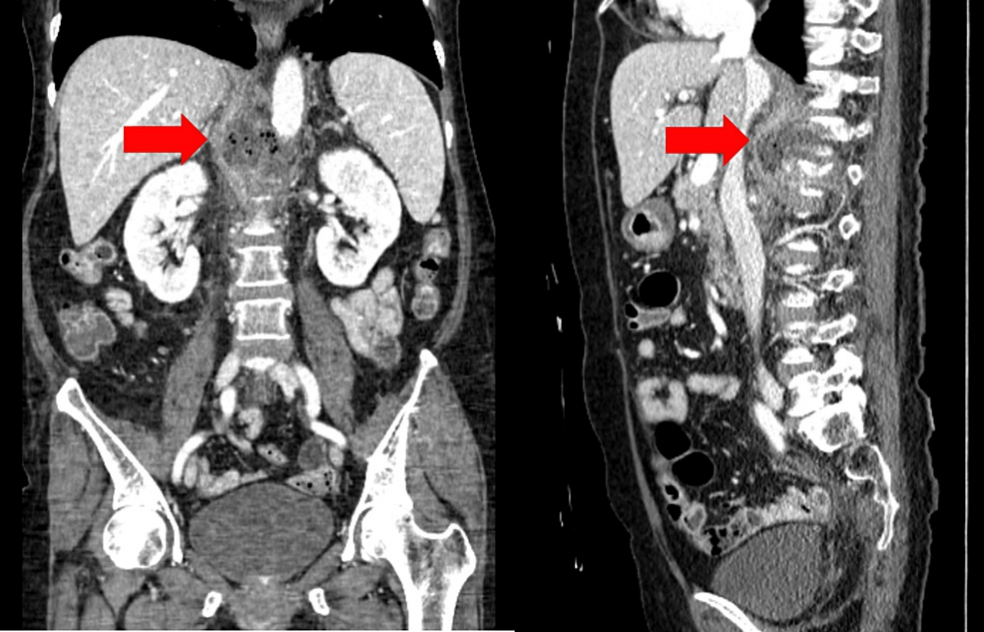
Abdominal and pelvic computed tomography coronal, sagittal view: abscess pocket from the T11 to L2 level (red arrows).

**Figure 3:**
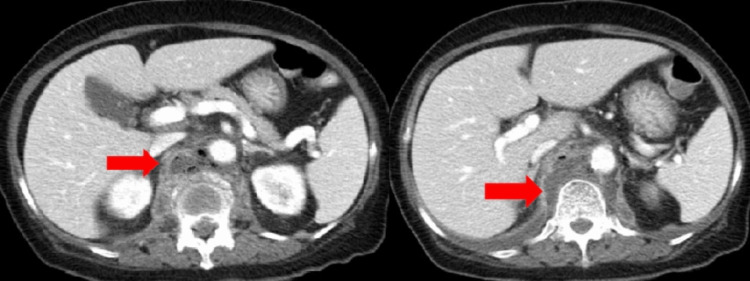
Abdominal and pelvic computed tomography axial view (L1 level): abscess pocket (red arrows).

**Figure 4:**
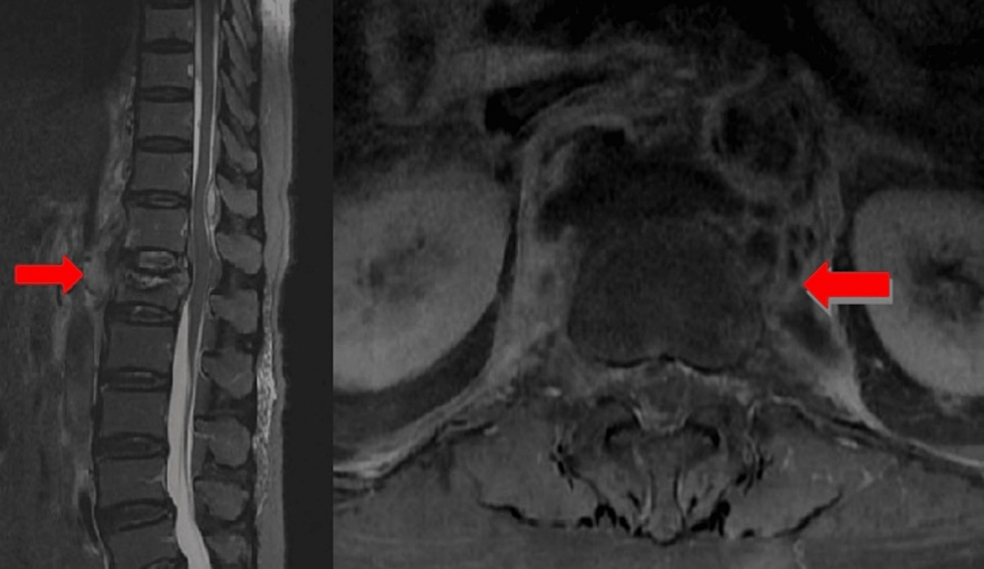
T2 weighted, fat suppressed magnetic resonance images: prevertebral and paravertebral abscess at T10-L1 level (red arrows).

Plain radiography of her lumbar spine showed a wedge-shaped compressive deformity on the L1 vertebral body (Figure 1). On abdominal and pelvic computed tomography (APCT) (Figures 2-3) and T2-weighted magnetic resonance imaging (MRI) (Figure 4), the lumbar spine showed high signal intensity from the T10 vertebra to L1, suggestive of spondylitis with a paravertebral abscess (size 6.2 × 2.6 × 10.1 cm^3^) involving the psoas muscle. After a careful pre-operative evaluation, posterior decompression and posterior instrumentation were performed because of intractable back pain, an epidural abscess with cord compression, and progressive vertebral body collapse.

**Figure 5:**
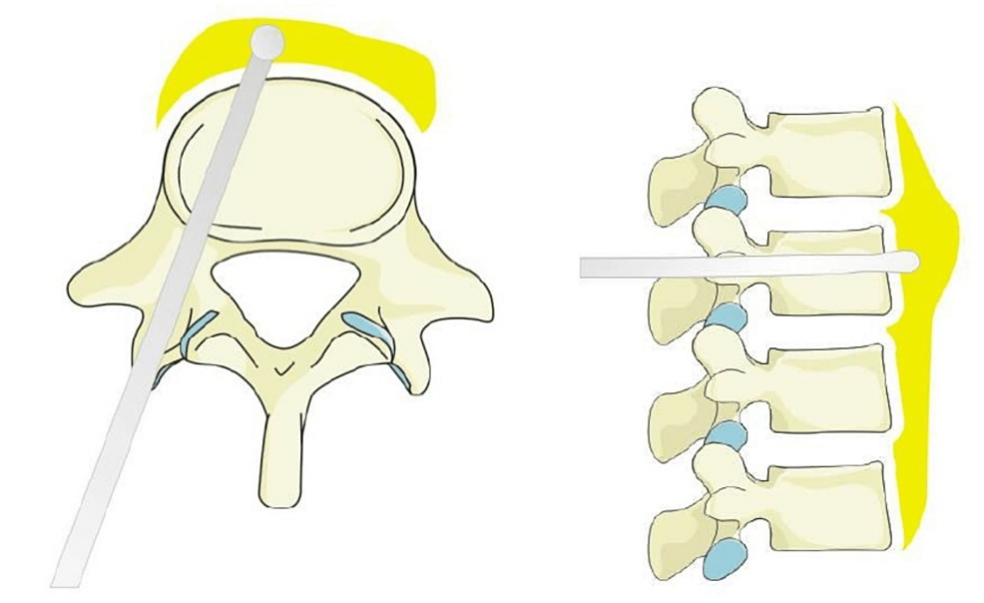
Transpedicular drainage procedure using the cannula. Penetrating and draining prevertebral abscess by advancing the cannula.

**Figure 6:**
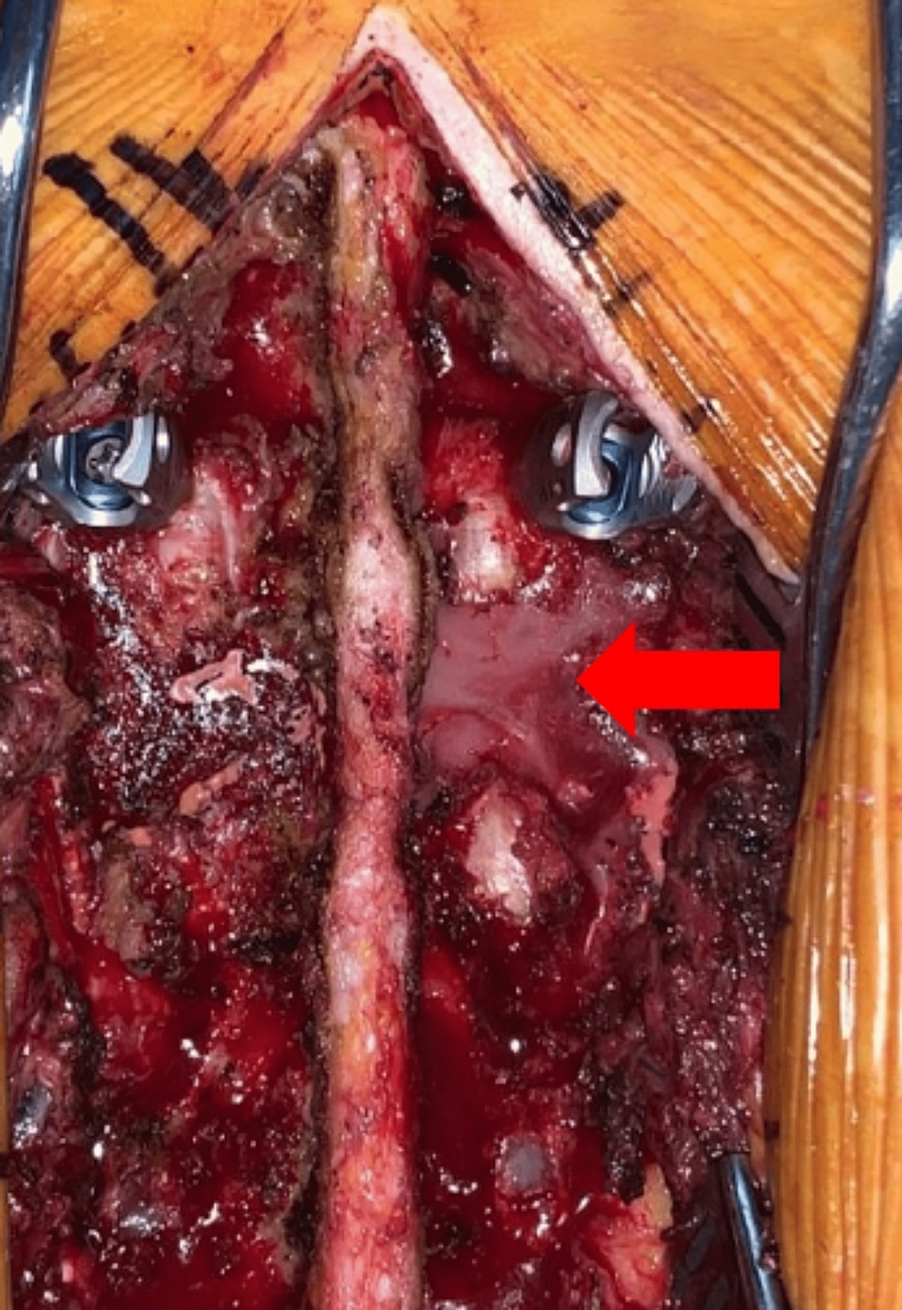
Intraoperative photograph after penetrating a pedicle. The red arrow indicates draining pus.

The patient underwent general endotracheal anesthesia. When she was placed in the prone position on the Jackson table, her vertebral height was partially restored. When the surgeon confirmed spine level using a c-arm image intensifier, vertebral height was partially restored. Dissection proceeded using a conventional posterior approach. When the surgeon made holes on the bilateral L1 pedicle with the Jamshidi needle, a large amount of pus oozed from the site and was extracted for culture studies. Then, the cannula was inserted through the hole and cleaned the cavity of the L1 vertebral body with 5 L of normal saline using transpedicular drainage technique (Figure 5). Irrigation flow was drained through the contralateral pedicle hole of the vertebra. After massive irrigation, the surgeon advanced the cannula carefully in order to remove the anterior abscess in front of the anterior vertebral body. After puncturing the anterior wall of the vertebra, more pus oozed along the pedicle, and massive irrigation was again applied (Figure 6). Using this surgical technique, we could remove the abscess effectively without an anterior corpectomy.

After laminectomy and removal of the ligament flavum of the T12 and L1 for decompression of the epidural space, posterior instrumentation was done by inserting pedicle screws and rods from the T11 to the L3.

The culture study from the intraoperative pus revealed S. gordonii, and any other pathogen was not identified. According to the dental department of our medical center, the cover screws of the implants used in the previous prosthetic treatment had loosened. The dental department diagnosed the patient with chronic gingivitis. Consequently, the screws were retightened, and an incision and drainage procedure were performed.

**Figure 7:**
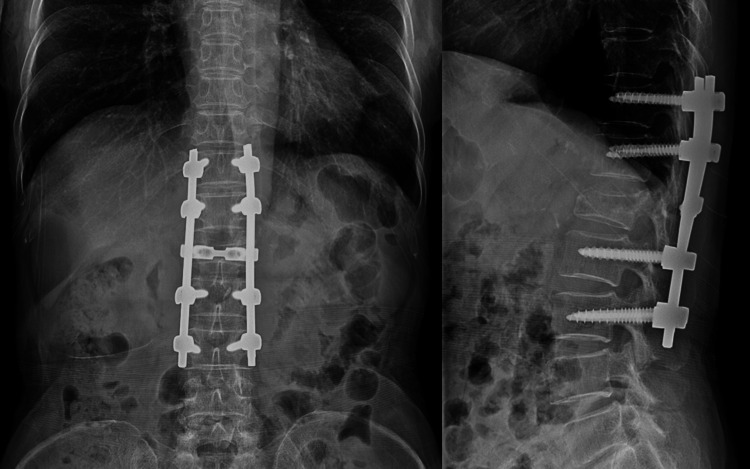
Plain radiographs 18 months after the surgery.

Upon arrival to our medical center, empirical antibiotics, including vancomycin, ceftriaxone, and metronidazole, were immediately applied. After receiving the results of an antibiotic susceptibility test, ceftriaxone monotherapy was applied for 24 days. One month after the surgery, the patient’s C-reactive protein level completely normalized, and her back pain was relieved enough that she was able to walk using a walker. She took oral moxifloxacin for two months after discharge. She is currently undergoing outpatient follow-up for 18 months, and the operation site is well-maintained without complications according to plain radiography examinations (Figure 7).

## Discussion

Infectious spondylitis usually occurs after direct inoculation or hematogenous spread via arterial and venous routes. The most common primary origin is the genitourinary system (17% of infections), followed by soft tissue infection (13%), hematogenous spreads (5%), and gastrointestinal tract (5%). Sometimes, it can be caused by intestinal infections, dental extractions, and hemodialysis [7]. Pyogenic spondylitis caused by *S. gordonii* has been rarely reported after a compression fracture of the vertebral body [8,9]. To the best of our knowledge, this is the first case of a surgical treatment used to isolate pyogenic spondylitis without other organ infections such as endocarditis or lung empyema caused by* S. gordonii* originating from the oral cavity.

In this case, the oral cavity was assumed to be the origin of the spinal infection because the patient had chronic periodontitis after a tooth extraction. No other suspicious sites for infection were identified. Furthermore, *S. gordonii* was the only pathogen cultured from specimen obtained during surgery, although it inhabits the oral cavity. Therefore, the authors conclude that the oral pathogen might be the cause of infectious spondylitis in this case.

Many studies have suggested that poor oral hygiene in elderly patients may cause aspiration pneumonia or periprosthetic joint infection. In the same vein, oral cavity infection can be a source of infection leading to systemic complications. Romagna et al. [10] have reported that in a study of 41 patients with infectious spondylitis, nearly one-third had oral cavity infections, indicating infectious spondylitis as a potential consequence of periodontitis. Advancing to infectious spondylitis from simple chronic periodontitis is uncommon. However, In this case, the patient’s general condition played a key role in the dramatic systemic transition. At the time of admission, the patient’s blood glucose level was 325 mg/dl with an HbA1c level of 9.3, indicating that diabetes was not well-controlled and the patient was vulnerable to infectious disease.

*S. gordonii *is a gram-positive, non-motile, α-hemolytic bacterium in the *Streptococcus sanguinis* group of *viridans streptococci* [11]. It primarily inhabits the oral cavity of humans and animals as part of the commensal flora. It is also an opportunistic pathogen that can cause a variety of diseases. As an initial colonizer on the tooth surface, *S. gordonii* can co-aggregate with several other oral microorganisms, contributing to the development of periodontal disease and caries [12]. When platelets aggregate in the cell wall of* S. gordonii* via mediators such as serine-rich glycoprotein and GspB, *S. gordonii *can spread in the blood stream and cause inflammatory reactions such as endocarditis, septic arthritis, and pyogenic spondylitis [13].

Infectious spondylitis usually occurs after direct inoculation or hematogenous spread via arterial and venous routes. The most common primary origin is the genitourinary system (17% of infections), followed by soft tissue infections (13%), hematogenous spreads (5%), and the gastrointestinal tract (5%). Sometimes, it can be caused by intestinal infections, dental extractions, and hemodialysis [7]. Pyogenic spondylitis caused by S. gordonii has been rarely reported after a compression fracture of the vertebral body [8,9]. To the best of our knowledge, this is the first case of a surgical treatment used to isolate pyogenic spondylitis without other organ infections such as endocarditis or lung empyema caused by S. gordonii originating from the oral cavity.

In this case, the oral cavity was assumed to be the origin of the spinal infection because the patient had chronic periodontitis after tooth extraction. No other suspicious sites for infection were identified.

Furthermore, *S. gordonii* was the only pathogen cultured from a specimen obtained during surgery, although it inhabits the oral cavity. Therefore, the authors conclude that the oral pathogen might be the cause of infectious spondylitis in this case.

Many studies have suggested that poor oral hygiene in elderly patients may cause aspiration pneumonia or periprosthetic joint infections. In the same vein, an oral cavity infection can be a source of infection leading to systemic complications. Romagna et al. [10] have reported that in a study of 41 patients with infectious spondylitis, nearly one-third had oral cavity infections, indicating infectious spondylitis as a potential consequence of periodontitis. Advancing infectious spondylitis from simple chronic periodontitis is uncommon. However, in this case, the patient’s general condition played a key role in the dramatic systemic transition. At the time of admission, the patient’s blood glucose level was 325 mg/dl with an HbA1c level of 9.3, indicating that diabetes was not well-controlled and the patient was vulnerable to infectious disease.

*S. gordonii* is a Gram-positive, non-motile, α-hemolytic bacterium in the *Streptococcus sanguinis* group of Viridans streptococci [11]. It primarily inhabits the oral cavity of humans and animals as part of the commensal flora. It is also an opportunistic pathogen that can cause a variety of diseases. As an initial colonizer on the tooth surface, *S. gordonii* can co-aggregate with several other oral microorganisms, contributing to the development of periodontal disease and caries [12]. When platelets aggregate in the cell wall of *S. gordonii* via mediators such as serine-rich glycoprotein and GspB, *S. gordonii* can spread in the bloodstream and cause inflammatory reactions such as endocarditis, septic arthritis, and pyogenic spondylitis [13].

In 2017, Dadon et al. [9] published the first two cases of spondylitis caused by *S. gordonii*, both of which were accompanied by infectious endocarditis. In 2019, Nakamura et al. [8] reported a case of empyema and pyogenic spondylitis diagnosed almost immediately after periodontal debridement. In both reports, pyogenic spondylitis was cured using conservative treatment. The current protocol calls for the intravenous administration of antibiotics for at least four weeks.

When intravenous antibiotics cannot penetrate into an abscess, inflammatory exudate, or intervertebral disc, surgery is performed to remove the infected lesion. Especially, in order to treat the prevertebral abscess and infected vertebral body, anterior corpectomy and fusion using an anterior approach had been the preferred surgical technique instead of transpedicular drainage and fixation [14,15].

In our case, surgical treatment had to be performed because of the unendurable pain and progression of an epidural abscess and vertebral body collapse. As mentioned, transpedicular irrigation and drainage were used as surgical treatments. Noting the abscess in front of the vertebral body on imaging studies, the surgeon directed drainage by inserting a cannula through the anterior cortex of the vertebral body. The patient achieved a good prognosis by having the abscesses around and in the vertebral body removed without a corpectomy.

This case study has some limitations. First, we could not confirm *S. gordonii* as the main cause of chronic periodontitis as specimens from the patient’s oral cavity and blood were not obtained at the time of dental treatment; hence, no culture test was conducted. If the bacteria identification test had been conducted to confirm the match, it would significantly increase the validity of our argument that periodontitis after tooth extraction treatment was the cause of her hematogenous spinal infection. Nevertheless, considering the type of organism obtained from the operation, the oral cavity is a possible source. Second, postoperative imaging studies could not directly confirm the results of the prevertebral abscess drainage. During surgery, an abscess at the psoas muscle level was drained by puncturing the anterior wall of the vertebra. However, there was no direct method to quantify the amount of abscess removed. Despite these limitations, the transpedicular drainage method offers several advantages as an alternative to transitional surgical drainage in treating pyogenic spondylitis: it is easy to apply for drainage, less invasive, and increases the effectiveness of antibiotic treatment.

## Conclusions

In patients with uncontrolled diabetes or another immunocompromised status, a minor infection from a dental treatment may progress to a serious spinal infection. Hence, physicians must always be mindful when treating these patients. We present the case of a 76-year-old female hospitalized due to infectious spondylitis, presumably caused by an oral pathogen that spread to her bloodstream during a dental procedure. Based on the patient's clinical symptoms, the progression of the vertebral fracture, and blood test results, surgical intervention was inevitable. After a successful surgery, the patient's clinical symptoms dramatically improved, and she had no problem carrying out her daily life. The present case calls for attention to infectious spondylitis that could stem directly from the oral cavity rather than from other organ infections, such as endocarditis or lung empyema, and be effectively treated with transpedicular drainage without corpectomy.
